# Discontinuance of the British American Medical Journal

**Published:** 1863-02

**Authors:** 


*Discontinuance of the British American Medical Journal,*—It is with deep regret we notice the discontinuance of the *British American Medical Journal* with the issue of December, 1862. This Journal has been one of the most ably conducted of our exchanges, and we are mortified in our feelings of pride in the profession of British America, that this Journal should be allowed a discontinuance because "*so large a proportion of subscribers are unwilling to part with the subscription price.*"

